# Two Paradigmatic Waves of Public Discourse on Nuclear Waste in the United States, 1945-2009: Understanding a Magnitudinal and Longitudinal Phenomenon in Anthropological Terms

**DOI:** 10.1371/journal.pone.0157652

**Published:** 2016-06-16

**Authors:** Judi Pajo

**Affiliations:** Department of Sociology/Anthropology, Dyson College, Pace University, New York, New York, United States of America; University of Liverpool, UNITED KINGDOM

## Abstract

This project set out to illuminate the discursive existence of nuclear waste in American culture. Given the significant temporal dimension of the phenomenon as well as the challenging size of the United States setting, the project adapted key methodological elements of the sociocultural anthropology tradition and produced proxies for ethnographic fieldnotes and key informant interviews through sampling the digital archives of the New York Times over a 64-year period that starts with the first recorded occurrence of the notion of nuclear waste and ends with the conclusion of the presidency of George W. Bush. Two paradigmatic waves of American public discourse on nuclear waste come to light when subjecting this empirical data to quantitative inventorying and interpretive analysis: between 1945 and 1969 nuclear waste was generally framed in light of the beneficial utilizations of nuclear reactions and with optimistic expectations for a scientific/technological solution; by contrast, between 1969 and 2009 nuclear waste was conceptualized as inherited harm that could not be undone and contestation that required political/legal management. Besides this key finding and the empirical timing of the two paradigms, the study’s value lies also with its detailed empirical documentation of nuclear waste in its sociocultural existence.

## Introduction

### The Ontology of Nuclear Waste

At a glance, nuclear waste might appear to be simply inorganic matter, something of physical and chemical properties that arises out of natural reactions, which happen to be harnessed by humanity only in relatively limited ways and relatively only as of late. But nuclear waste is also, and importantly, a sociocultural phenomenon.

Acknowledging nuclear waste its sociocultural existence does not deny nuclear waste its material existence. As a fact of the material world, what we commonly refer to as nuclear waste consists of a range of byproducts of the exploitations of nuclear fission for human ends. Nuclear fission involves the splitting of atoms of radioactive elements through a process that typically hits those atoms with neutrons and causes them to release neutrons, radiation, and heat. The bulk of the heat and radiation that are released as atoms split are then channeled into uses such as electricity production, propulsion, or medical imaging. To be submitted to this process efficiently, especially for purposes of energy production, radioactive atoms, typically of uranium, are processed into what is called nuclear fuel. Such fuel is typically not fully decayed while fissioned in a nuclear reactor: after nuclear splitting slows past the point where it can be exploited efficiently, used fuel continues to decay and emit both radiation and heat. This decaying radioactive matter is one prime example of what is called nuclear waste. The intensity at which different types of spent nuclear fuel or other kinds of nuclear waste emit radiation and the time periods over which they do so vary from fractions of a second to tens of thousands of years.

At the same time, acknowledging nuclear waste its sociocultural existence is also not simply focusing on its original fact of arising out of the human exploitation of the material world. It is true that nuclear waste is a byproduct of culture: the exploitations of nuclear reactions from which nuclear wastes arise only commenced after the human discovery of radioactivity at the end of the nineteenth century; the initial employments of nuclear reactions in medical radiology were followed by military uses in World War II and subsequently through the Cold War; the exploitation of nuclear fission for civilian energy production, which generates much of the bulk of nuclear waste, really took flight only after the end of World War II. Yet, cultural and rooted in human endeavor as these origins are, as origins of nuclear waste they are still but the origins of a particular state of material matter which remains material matter.

Nuclear waste is a sociocultural phenomenon aside from its material existence and separately from its material existence. Though nuclear waste’s sociocultural existence is predicated upon nuclear waste’s material existence, nuclear waste the sociocultural phenomenon does not consist of radioactive matter but it is instead manifested in concepts that take place in discourse: importantly, shared concepts in public discourse. As such, nuclear waste is a lively conceptual and discursive phenomenon that exists in and with other concepts and discursive phenomena in settings that include, among others, the shared or public culture of the US American society. The launching premise for the present study is that this existence of nuclear waste calls for an anthropological understanding.

### Literature Backgrounds

A substantial body of academic literature has sought to tease apart the specifically not-physical and not-chemical but political and legal and cultural aspects of nuclear waste. The coverage of such scholarship includes the contexts of energy production in which nuclear waste typically comes up as a topic. For example, a few years ago Richard Burleson Stewart and Jane Bloom Steward published an extensive analytical study of the complex legal contexts of, and the key processes of policymaking on nuclear waste in the United States [1]. Earlier, J. Samuel Walker, a historian with special access to government records, had chronicled the history of federal plans for a national repository for high-level nuclear waste at Yucca Mountain in Nevada that grew into an issue of key symbolic as well as practical importance [2]. The realization that public culture and public discourse, which are topical cores of the present study, concern the broader nuclear topic well beyond nuclear waste, underlies Spencer R. Weart’s studies that seek to account for the “fear” of nuclear in the United States [3].

In addition to these as well as other book-length studies on the topical domain, this researcher reviewed about three dozen related peer-reviewed article-length studies in the process of designing and carrying out the project outlined here. These articles appeared in journals across the social sciences from the late 1970s, mostly after the 2000s. Further broadening the scope of the books outlined above, these articles typically explore more specific themes of significance to nuclear waste such as attitudes and risk perceptions [4–8]; public acceptance [9]; opposition and environmentalism [10–11]; sites and the siting process [12–16]; ethics, responsibility, justice [17–19]; safety concerns [20]; security concerns [21]; property values [22]; other management and policy issues [23–30]; and methodological innovations [31–32].

The theme of risk and the theme of uncertainty may be seen as providing the larger framework under which much of this literature can be meaningfully understood. (And this turns out to fit well with this paper’s findings and analysis with regard to the second paradigm.) In the literature, important dimensions of risk appear to associate with science and technology, as discussed for example in investigations of significant cases such as Yucca Mountain, where scientific and social/cultural uncertainties are related to new approaches to managing nuclear waste [33]. Risk as insightfully theorized by Ulrich Beck [34–35] is indeed a broader theme that has inspired empirical investigations beyond the issue of nuclear waste and beyond the borders of the United States across cultures and societies [36].

### The Case for Anthropological Understanding

One thing that appears to be commonly shared across this range of scholarship, despite the rich diversity of the specific foci or the conclusions that individual studies reach, is that the very concept of nuclear waste is accepted as no more than a mere reference to the material nuclear waste. So, while illuminating numerous sociocultural aspects of it, the literature also tacitly equates nuclear waste in its sociocultural existence to its namesake of physical matter. Yet the sociocultural existence of nuclear waste extends well beyond that of a mere signifier: it involves the perspectives from which nuclear waste is understood, it involves which aspects of its natural existence are highlighted and which are dismissed, it involves which assessments are laid upon which relations to which historical or political or social or cultural events. Arguably, it eventually involves which checkbox is checked when deciding the future of nuclear waste siting facilities or even the future of energy production.

That is not to suggest that because of typically not investigating the concept of nuclear waste on its own right the selection of studies cited above is in some way flawed or diminished in importance. In this researcher’s view, all of the studies cited above, among others in a growing body of literature, remain both informative and insightful. Instead, the present project built on the premise that this solid body of knowledge would be enhanced if we also investigated the sociocultural existence of nuclear waste on its own right.

To such an investigation, an anthropological approach appeared to be particularly promising. In a canonical tradition of studying culture, anthropologists have enriched the general understandings by questioning commonly held cultural constructs through empirical research or even by challenging widely-held perceptions through often politically bold interpretation [37–39].

One immediate focus for an investigation of nuclear waste’s sociocultural existence may be the very current identity of the concept. Tacit acceptance of the concept of nuclear waste as simply a linguistic or discursive signifier suggests some kind of timeless constancy to it, an unchanging identity similar to that of the actual nuclear reactions from which the physical nuclear waste arises. As a participant in contemporary American culture, one is likely to associate nuclear waste foremost with grave hazards to human health and to the earth’s ecosystems. And one is likely also to point out that there are good reasons for that association: to date, no way of eliminating the hazardous radiation of nuclear waste is known to exist; none of the known disposal methods can promise beyond dispute to keep nuclear waste from entering the human food chain and eventually human bodies. But was the common understanding of nuclear waste always so? Or could it be that, despite the unchanged natural laws governing nuclear reactions and radioactive decay, the public concept of nuclear waste transformed over time rather like other phenomena of the discursive world? Then, if so, how so?

## Research

### Designing the Empirical Study

As a shared sociocultural phenomenon, nuclear waste may be present in any location where people are present who engage in shaping it by putting into words their thought about it; its setting is as broad and as deep as that of shared public life. So while an anthropological approach promises to enhance our understanding of nuclear waste in its sociocultural existence, designing a data strategy for carrying out an empirical investigation presents two key challenges: magnitude and longitude.

The core of the sociocultural anthropology tradition, as it is commonly taught and understood, lies with the researcher personally going to an unfamiliar human community where the researcher spends a substantial amount of time, often in the range of several consecutive months. Known as participant observation or ethnographic fieldwork, this methodology consists of observing the broadest possible range of daily practices, in which, and for as much as possible, the researcher also personally participates. The purpose of such effort is to achieve an understanding of the world from the community’s shared perspectives. The data collected through this traditional methodology consists mostly of a record of the researcher’s own observations and impressions as well as statements from exchanges and interviews with the members of the previously-unknown community that, ideally, becomes better-known over the course of this process. These textual records typically form the basis for subsequent analysis, interpretation [40], and sometimes even methodologically sanctioned “reflections” [41], which ultimately lead to and/or represent the sought understanding of the studied community’s shared perspectives on the world.

In terms of geographical size and population size, however, the United States provides a stark contrast to the places where anthropologists traditionally carry out their investigations. Furthermore, as nuclear waste has been a topic of public engagement in America for decades, its unfolding over time represents a significant dimension of nuclear waste as a sociocultural phenomenon; this temporal dimension simply cannot be investigated by a straightforward employment of conventional research methods.

To overcome these challenges, the researcher made an adaptation to the key methodological convention of sociocultural anthropology. It was based on the researcher’s recognition of the American public discourse on nuclear waste as a case for which extensive records clearly existed. Following this recognition, the researcher further identified the archive of the New York Times as a useful source of such records for the American public discourse—a source that could proxy for the fieldnote and interview records of a conventional ethnographic research project. (One of the journal’s anonymous peer reviewers raised the concern that proxying archival data for ethnographic fieldnotes disqualifies the methodology as not ethnographic, and that no prior literature justifies this study’s approach. The author contends that, though doing something that has not been done before indeed amounts to innovation, the search for novelty in anthropological methodology is not at all new. Many other anthropologists have moved beyond the traditional ways of doing ethnography. In fact, two of the most influential figures in contemporary anthropology, Arjun Appadurai and George Marcus, have theorized on such innovation. For example, Marcus’s writing on the emergence of multi-sited ethnography in the Annual Review of Anthropology is a landmark of contemporary anthropology [42]. Appadurai has discussed the emergence of “scapes” as grounds for anthropological/ethnographic research—scapes such as ethnoscapes, technoscapes, finanscapes, mediascapes, and ideoscapes, that are clearly not the sites of traditional fieldwork [43].)

One advantage of this conceptualization is that the publication records of a leading media organization such as the New York Times, which are professionally archived and maintained and made publicly available, represent not mere months of observation by one individual researcher but decades of unfolding action by a multitude of observers and participants. In addition, while the record of the American public discourse on nuclear waste is certainly broader than that of the source selected here, limiting the source of data collection to the records that are available from the archives of the New York Times presented two important advantages. One: a clearly defined, comprehensive, and well maintained digital archive would allow for good control in sampling as well as for replication. Two: as the United States’ newspaper of record since the early days of exploitation of nuclear reactions, the New York Times may, arguably, have not simply recorded the American discourse on nuclear waste but it may also have played a part in shaping the sociocultural existence of nuclear waste, amongst other sociocultural concepts.

### The Dataset

The first step in creating the dataset was to narrow the broad range of reporting published in the New York Times on nuclear topics to only those items that included in their headlines the keywords “atomic,” “radioactive,” or “nuclear” in conjunction with the keyword “waste.” This step led to a logical further narrowing of the sample to the 64 years period of 1945–2009: while the digital archives of the New York Times start with the year 1851, the first records that include any one of the keyword pairs listed above appear only in 1945; as the number of sampled hits appeared to fluctuate by year, hinting at the possibility of correspondence between such fluctuations and larger political contexts, the researcher decided to meaningfully limit the sample to the conclusion of the presidency of George W. Bush, thus leaving out the years of the presidency of Barack H. Obama that were/are presently unfolding. The time-limited query revealed that 21,747 headlined items published in the New York Times between 1945 and 2009 contained in their headlines one of the keywords “atomic,” “radioactive,” or “nuclear.” Of these, only 693, or roughly 3%, also included in the headline the keyword “waste.” The New York Times archive classified these 693 items, which constitute the present project’s dataset, as articles, letters to the editor, front page articles, editorials, and other: 566 are articles, 57 are letters to the editor, 50 are front page articles, 18 are editorials, 2 are other formats.

### Discourse Analysis and Ethnographic Portrayals

Given that “discourse analysis” is often used broadly and refers to practices that can range from exegesis of entirely qualitative data to entirely computerized processing of quantitative data, a specification is in order: the analytical procedure employed in this project may be reasonably described as discourse analysis that is systematic, qualitative, and interpretive. In order to guide and inform the interpretations outlined in following, the researcher conducted a measure of counting and comparing of frequencies for a selection of key notions that occurred in the textual records in the dataset. However, the paper’s conclusions were ultimately reached on basis of cataloging and qualitatively interpreting the meanings and perspectives that, in the researcher’s understanding, underlay the words of the written records. At the foundations of this analytical framework lie certain insights from the cognitive and interpretive traditions in social and cultural anthropology where making sense of communication have a long and venerable tradition with highlights ranging from the structural analyses and theorizings of Claude Lévi-Strauss’ [44–45] to the cognitive theories of George Lakoff and Mark Johnson [46] as well as of Claudia Strauss and Naomi Quinn [47]. In addition, the researcher was also influenced by Michael Burton’s work on Micronesian households [48–49]; Sherry Ortner’s plotting of the evolution of cultural understandings [50]; and Willett Kempton, James Boster, and Jennifer Hartley’s employment of similar analytical frameworks for mapping the different environmental values in American culture [51]. In addition, to convey the sociocultural existence of nuclear waste in richer ethnographic style, the researcher adapted a portrayal strategy that aims at generating texture or “thick description” through citing for as much as possible the words of the informants [50,52]: in the present paper, selections of segments from the articles in the database substitute for such informant quotes.

### Validity and Limitations

The researcher recognizes that notwithstanding the merits of the digital archive of the New York Times as a proxy for ethnographic fieldnotes or key informant interview records, alternative promising sources exist beyond the one used in this project. The archives of other major newspapers (such as Wall Street Journal, Los Angeles Times, Chicago Tribune, San Francisco Chronicle, Washington Post, etc.), or any amalgamation of records from smaller daily presses, or any amalgamation of records from other periodicals and publications, may be sampled and analyzed to falsify the findings of this present research and/or to further and to complement the insights into the sociocultural existence of nuclear waste in American public discourse that this present research has developed.

### Analysis and Findings

**Fig 1:**
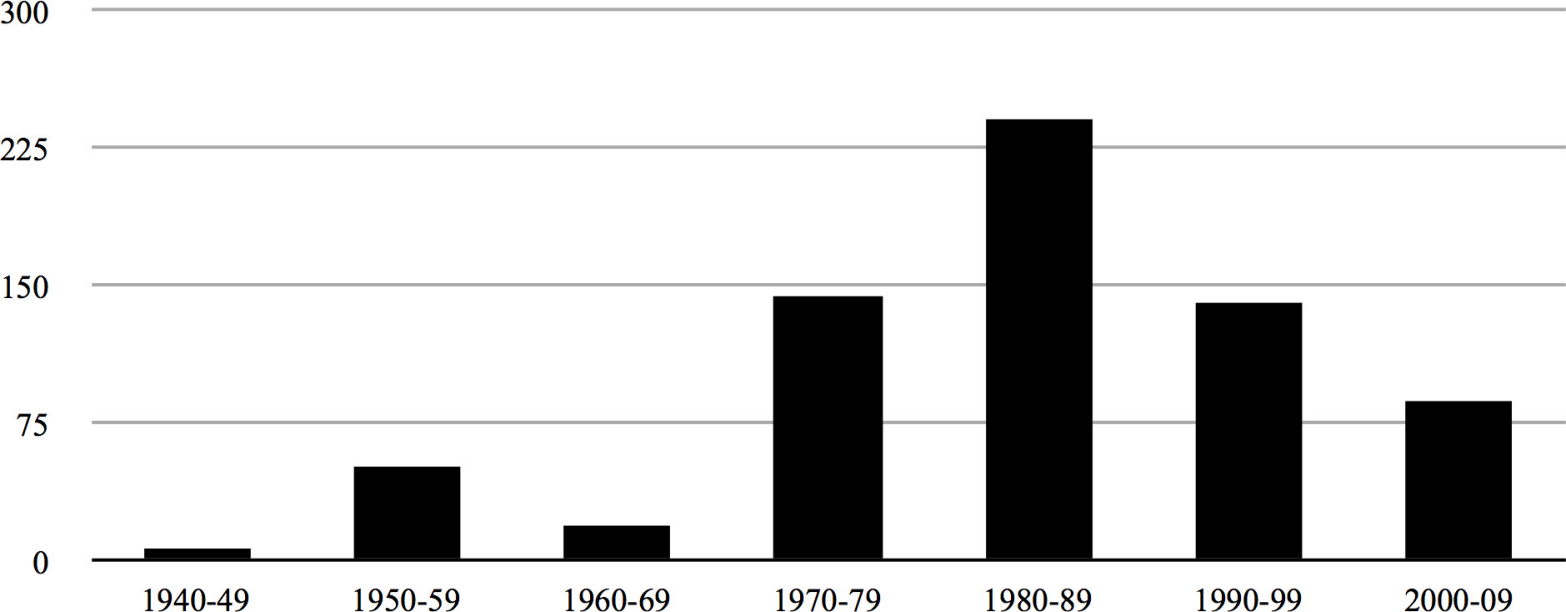
Total number of reports by decade.

**Fig 2:**
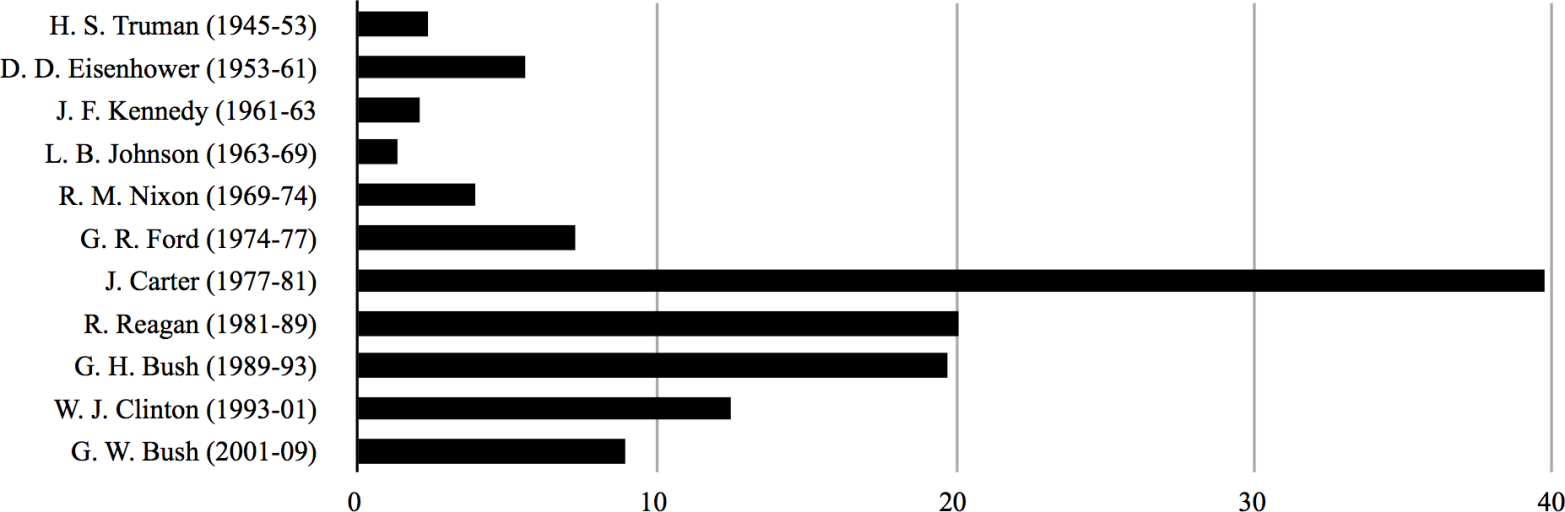
Annualized rate of reports by presidents.

The initial discovery popped out of simply plotting the distribution of the dataset samples in a bar chart organized by the decade in which they were published. This revealed a curve that peaks twice: first in the 1950s and then in the 1980s (Fig 1). A similar pattern was revealed when plotting the annualized rate of publication for articles on nuclear waste by presidency: in the second plotting, the first peak forms under President Eisenhower, 1953–1961; the second under President Carter, 1977–1981 (Fig 2).

Then the researcher familiarized herself with the items sampled in the dataset. In the typical or traditional employments of the anthropological method, though formal analysis and interpretation take place only after fieldwork has first taken place, the researcher becomes familiar with the data already over the course of conducting the research: the final analyses and interpretations are of the researcher’s own collections and recordings. In the case of this project, the researcher drew the dataset without such prior familiarity—though undoubtedly with what an anthropologist would call the “native understanding” that may reasonably be attributed to a member of contemporary American culture.

After the initial familiarization with the data, the researcher read closely each one of the 693 items in the dataset, proceeding in the chronological order of publication. This revealed a multitude of themes through which the public discourse, as recorded on the pages of the New York Times, conceptualized nuclear waste between 1945 and 2009. In order to define these conceptual frameworks, the researcher spelled out the underlying and often implied meanings that were conveyed in the language of the reporting, the modes of framing or representing issues, and then worked on systemizing these. Key among these foci and meanings and shared perspectives were the benefits of nuclear exploitation, the hazards of radioactivity, the methods for the disposal of nuclear waste, the inability to achieve safe containment, numerous health and environmental concerns, a variety of radioactive isotopes, scientific discovery, technology, sites of disposal, costs of disposal, the role and the actions of government, the national and the international players, political action, legal disputes, the environmental movement, the emergence of industry, as well as numerous identifiable individuals depicted in their actions and sometimes cited in their own words.

Already over the course of identifying these themes, and while subsequently reflecting on these findings, it became clear that the unfolding story of the recognition of nuclear waste as an issue of public importance and its subsequent evolution into an issue of political and legal disputes was captured and framed in public discourse from the angles of two key but strikingly distinct conceptual frameworks. Another important insight emerged at this point: these two underlying frameworks seemed to correspond to the two peaking waves in the distributions of the dataset. To a cultural anthropologist, it was as if studying two different communities: the 1945–1969 period expressed views that were strikingly different from those of the 1969–2009 period. To formalize these findings, and to help capture the wide sharing of the underlying meanings and perspectives, as well as the eventual replacement of the one set of such shared meanings and perspectives with another, the researcher used the concept of “paradigm,” as developed by Thomas Kuhn [53] and as used fruitfully across the social sciences. In the following, the paper outlines and then fleshes out in ethnographic style these two paradigmatic waves of American discourse on nuclear waste.

### The First Paradigm: “Science and Technology” of Nuclear Waste

Nuclear waste has been making headlines ever since 1945. New York Times reports that include in the headline some combination of the keywords “atomic,” “radioactive,” “nuclear,” and “waste” first appear shortly after the bombing of Hiroshima and Nagasaki. The body of reporting identified here as “the first wave” corresponds to the presidencies of Harry S. Truman (1945–1953), Dwight D. Eisenhower (1953–1961), John F. Kennedy (1961–1963), and Lyndon B. Johnson (1963–1969). A total of 77 items of reporting are distributed by presidency as follows: 19 reports under the Truman presidency, 45 under Eisenhower, 6 under Kennedy, 7 under Johnson.

While a distinct paradigm of nuclear waste disposal is clearly identifiable in this period, it is important to notice that public discourse on the nuclear topic between 1945 and 1969 appears to have centered not around the issue of nuclear waste disposal. Reading this sample of reports suggests that, instead, discourse on nuclear matters in this time revolved around the beneficial utilization of nuclear power in medicine, as well as for peace, prosperity, and energy production. While the reporting focuses on scientists exploring beneficial uses of nuclear energy, there is recognition that a byproduct of the beneficial uses is radioactive waste. Public health concerns of medical doctors find their way into the reporting, eventually followed by broader environmental concerns expressed by a variety of scientists. The reporting explores a range of specific problems with incumbent disposal practices. However, even then, a safe-disposal solution is sought from research and anticipated to be developed in the near future. Therefore, the researcher concludes that a key characteristic of the 1945–1969 paradigm underlying the discourse on nuclear waste is shared optimism about a near-future solution to the nuclear waste problem. In the following paragraphs the researcher thick-describes in more recognizably ethnographic style the public discourse on nuclear waste as represented in these reports, outlining the key features of the dominant paradigm and illustrating the analysis with a number of quotations from the data.

### The First Paradigm in Thick Description

One of the characteristics of public discourse to first strike a researcher reading these reports is the conceptualization of nuclear weapons primarily as a means of bringing peace. A September 9, 1945 report, titled “Atomic Bombing of Nagasaki Told By Flight Member,” illustrates this: “‘Think this atomic bomb will end the war?’ he [Sergeant Curry] asks hopefully. ‘There is a very good chance that this one might do the trick,’ I [William Laurence] assure him, ‘but if not, then the next one or two surely will. Its power is such that no nation can stand up against it very long.’” In this record, clearly, the bomb was the conceptualized primarily in terms of its beneficial consequences, namely peace, rather than the immediate destruction that it caused.

The optimism about the benefits of exploitation of nuclear reactions revolved around not only peace but also the prosperity that would arise from nuclear energy production. Interestingly, while it was the Truman administration that introduced the keyword “energy” into the public discourse on the nuclear topic as it created the Atomic Energy Commission, it was under the Eisenhower administration that the effort to produce nuclear energy for civilian purposes really started. A January 27, 1954 report, titled “Electricity is Made from Atomic Waste” even links nuclear waste itself with the beneficial use of nuclear reactions for energy production. This report relates an incident involving David Sarnoff of the Radio Corporation of America: “[Sarnoff] showed how the new atomic battery could be made to perform useful work. Hooking it to an old-fashioned telegraph key, the man who began his career as a telegraph operator tapped out a message. It was the first message ever sent with electricity obtained by the direct conversion of atomic energy. It read: ‘Atoms for peace–.’” “Atoms for peace” happens to be the name of the policy to share uranium with foreign nations for peaceful purposes that was put in place by the Eisenhower administration. In this context peaceful purposes really meant prosperity.

As a report titled “Radioactive Waste: Bacteria as a Means of Purifying Material” recognizes, public discourse is well aware that radioactive waste is a problem in need of a solution: “This solution of the problem presented by radioactive wastes is not yet in the practical stage.” This February 13, 1949 report was published in the “Science in Review” section of the paper. It clearly shows that scientific discovery is where that solution was expected to come: “Experiments have been made at Los Alamos which indicate, according to Dr. Ruchhoft, that a two-stage activated sludge process would probably remove all but 1 percent of the plutonium in the wastes—not enough to contaminate water or soil dangerously.” Five years later, that solution was yet to be found. A February 21, 1954 report, titled “Notes on Science,” recounts another instance of scientific effort to reduce the atomic waste pile: “At the University of Texas experiments have shown that the radioactivity of these wastes in liquid form can be taken up by algae, whereupon the algae are removed by rotary vacuum filters. In this way the volume of radioactive liquid which must be retained can be greatly reduced.”

The anticipated solution would be technological and/or industrial in nature; it would, furthermore, involve reprocessing radioactive nuclear waste into a reusable resource. An immediate area for such reuse would be the production of nuclear weapons. An August 1, 1950 report, titled “Weapons Using Radioactive Poison Pushed by Atomic Energy Board,” establishes this link: “Such wastes may turn out to have industrial value, or may be used in the fabrication of weapons, according to Dr. Walter Claus […who…] declined to conjecture whether such substances could be delivered on an enemy in a mist or dust.” Weapons, however, were not the only area envisioned. Already in its title, a February 11, 1951 report speaks of other ones. Titled “Dangerous Radioactive Wastes, which are now Buried, May Have Many Industrial Uses,” this report expresses the underlying idea of reuse clearly: “A possible solution of a major problem of the atomic age–the disposal of large quantities of dangerously radioactive wastes–was suggested by Dr. F. C. Henricques Jr. of Boston. According to him many industrial uses such as the sterilization of food and pharmaceuticals may be developed from fission products which are now buried.” A January 27, 1954 report further implies that waste used to generate electricity ceases to be waste: the report is titled “Electricity is Made from Atomic Waste.” Three years later, on January 30, 1957, a report titled “Wide Uses Promised for Tiny Atomic Battery,” advances this thread: “The [battery] cell uses as its basic material a radioactive waste byproduct of nuclear reactors, prometheum 147. It produces energy for almost limitless periods of time and requires virtually no shielding against radiation.”

While safe disposal solutions were being pursued in the science and technology front, the accumulating radioactive waste had to be put somewhere. Public discourse displays intense interest in the ways in which nuclear waste was actually disposed of. A December 23, 1949 report, titled “U.S. sets up public health branch to plan fight on atomic hazards” indicates that the practice of burying nuclear waste was initially viewed as normal and safe: “Residents of New York City and its environs were assured that by restriction of the operation of the atomic ‘pile’ of the Brookhaven, L.I., plant to certain wind conditions and by the burial of ‘slugs’ or metal containers of irradiate products, the area in general and the near-by water supply sources were protected.” This underlying view of safety would soon change, however. An August 7, 1950 report, titled “Brookhaven Guards Lives: Provides Close Check on Waste Material From Atomic Pile” uses the same site, Brookhaven National Laboratory, to reveal the practice of burial as more complicated than one might assume from reading the quote above: “Used radioactive material, the laboratory said, is collected in small tanks after it goes into the drains. If it ‘cools off,’ it goes on into sewers. But if it remains dangerously radioactive, it is sealed in permanent tanks. Any accidental seepage, the laboratory added, can be detected quickly and corrected before harm is done. Gas used to cool off the atomic pile is highly diluted before being released into the air. And just to be safe, the laboratory said, it checks the air from time to time.” In other words, burying nuclear waste did in fact mean not just burying: in addition to burying, nuclear waste was also flushed into sewage and released into the air.

This would have started to make nuclear waste disposal problematic in the public view. A September 17, 1950 report from the “Science in Review” section of the paper, titled “How to Dispose of Deadly Radioactive Wastes is a Difficult Problem for Atomic Plants,” makes this rather visual: “If a rat were trapped and killed at Oak Ridge, Hanford or Los Alamos, the disposal of his carcass, if it were strongly radioactive, would present a little problem. The carcass cannot be buried (…). Nor can it be thrown into the garbage can to be dealt with like any city waste. This department does not pretend to know just what happens to a dead radioactive rat, but it suspects that the carcass is burned and the ashes added to other radioactive wastes that must be disposed of in some appropriate way.” Further on, this same report indicates that there would have been serious questioning. The “newspaper men in Washington,” following up on a semi-annual report of the Atomic Energy Commission, received the following answer by one Dr. Walter Claus of the Commission: “When necessary, the wastes are buried for so long a period that they turn into harmless elements.” Implied here is that the public discourse has entered the theme of danger: there is recognition of the presence of harmful elements as well as of the government scientists’ effort to turn them harmless.

At one point, the oceans emerge as the frontier for disposal. On April 15, 1948, a report titled “Atomic Waste Pile Menace to Safety” already informed of a meeting of scientists to discuss “dumping at sea;” it is mentioned that “simple dilution not enough–mixing in concrete blocks and sinking found safer.” A July 13, 1957 report, titled “Nuclear Waste Shipped,” informs the readers, now without any comments, of the dumping of radioactive waste 150 miles southeast of New York City. And within less than two months, the tone has become more engaged: the title of a report from September 5, 1957 reads: “‘Hot Cargo’ Gets One-Way Sea Trip.” The ocean dumping reported here is closer to where people live: “about twenty-seven miles off the Massachusetts coast.” A decade later, on December 28, 1967, the readers learn that “[e]leven thousand tons of solid radioactive waste were dumped in the Eastern Atlantic last summer (…). Solid wastes from nuclear centers in five European countries–Britain, France, West Germany, Belgium and the Netherlands–were packed in containers and discharged at a depth of 16,400 feet.”

Though pursued as an alternative to land disposal, ocean disposal too was soon associated with concerns about health. Even though these concerns do not appear to be a main theme of public discourse, they do come to the surface. A July 29, 1950 report links the practice of ocean disposal of radioactive waste to human health: “If fish ate the material, scientists fear it might find its way into food used by humans.” A May 23, 1966 report titled “No Hazard Found in Atomic Wastes” does suggest however the possibility of hazard by emphasizing the time dimension of safety: “World Experts Call Dumping into Seas No Peril Now.” The report goes on to discuss a fear of contamination and the investigation of radioactivity in sea life in Atlantic and Pacific Oceans.

With both land and water thus appearing problematic, ideas of blasting nuclear waste into outer space are entertained. A July 29, 1950 report, titled “Atomic ‘Cemetery’ Needed for Waste,” envisions rocketing radioactive waste into the extraterrestrial space. The report recognizes the gravity of the problem of radioactive waste, depicts it as lethal, depicts storage as costly, and suggests “to cast the atomic waste coffins in heavy concrete and lower them off into one of the great deeps of the ocean or shoot them off the earth in a rocket that would become a satellite in outer space.” In the context of earlier reports of radioactivity spread into soil, air, water, the coffin imagery reveals the implied desire for a disposal that is final.

By 1962, the reprocessing of radioactive waste was technologically feasible on an industrial scale. A July 6 report from that year, titled “Private Plant for Reprocessing of Atomic Fuel Planned Upstate,” tells in technical detail of New York Governor Nelson Rockefeller’s plans for a state-owned and privately operated reprocessing plant: “The fuel reprocessing that the company will engage in is like sifting the ashes out of coal. Nuclear fuel has to be removed from reactors when only 2 per cent of its energy has been used because the ash or waste prevents it from burning. Included in the nonreusable waste will be a number of atomic by-products like plutonium, and strontium 90 and cesium 137. Strontium 90 and cesium 137 are used in the making of atomic batteries for satellite communications systems and remote automatic weather stations in the Arctic and Antarctic.”

However, all these efforts, whether for safe disposal or reprocessing, failed to produce the desired final solution to nuclear waste. An April 15, 1948 report, titled “Atomic Waste Pile Menace to Safety,” already draws attention to the hazard and the ubiquity of radioactive waste: “The populations of large areas may find the air that they breathe, the food they eat, the water they drink, and perhaps anything they that touch contaminated with harmful quantities of radioactive material.” Nearly a decade later, a June 5, 1957 report, titled “U.S. combating Food Radiation,” makes explicit the insufficiency of any of the then-current methods for preventing the harm caused by radioactivity: “no adequate methods have yet been developed for getting rid of the wastes of the nuclear reactors expected to be built in the next twenty-five years.” Eventually, there is even effort to ameliorate concerns about the hazard of radioactive waste. A May 29, 1966 report, titled “Reassuring Report on Radioactive Waste,” first goes into the science of radioactive waste and then assures the readers that contaminated food and drink are not necessarily harmful: “As cooling water from the Columbia River flows through the Hanford reactor it is subjected to neutrons that attach themselves to atoms of various elements in the water, making them radioactive. Some of these so-called isotopes quickly decay back to inert forms. Others are long-lived, but do not enter into the life-process. They can impart an external dose, but they do not enter the body except as transients (in food or drinking water). Finally, there are those that are both long-lived and absorbed by living organisms.”

In all of these illustrations, indeed in the nuclear waste paradigm of 1945–1969, the action is carried out by the US government and its scientists, researchers, and inventors. At times, the US is shown in competition or cooperation with foreign nations. The foreign nations may also appear as actors on their own right. A May 22, 1949 report, titled “Atomic Waste Dumped at Sea,” focuses on the British: “The British are taking the radioactive waste (…) out to sea in sealed drums and dumping it several hundred miles southwest of the British Isles in 2,000 fathoms.” A December 28, 1967 report informs the readers that solid radioactive wastes “from nuclear centers in five European countries–Britain, France, West Germany, Belgium and the Netherlands–were packed in containers and discharged at a depth of 16,400 feet.” A July 30, 1967 report, titled “Japan is Planning Atomic Ship in ‘70,’” informs that Japan will also be dumping radioactive waste at sea. A May 23, 1966 reports on the Russians: “[T]he Russians have officially opposed any discharge of atomic wastes into the seas. They argue that as nuclear plants become more numerous the oceans could become sufficiently contaminated so that sea life would become hazardous for consumption.”

### The Second Paradigm: “Political Economy” of Nuclear Waste

Nuclear waste continued to make headlines after 1969. Between 1969 and 2009, the New York Times reports that included in their headlines some combination of the keywords “nuclear,” “radioactive,” “atomic,” and “waste” appeared amidst reports on political and environmental protests. The body of reporting identified here as “the second wave” corresponds to the presidencies of Richard M. Nixon (1969–1974), Gerald R. Ford (1974–1977), James Carter (1977–1981), Ronald Reagan (1981–1989), George H.W. Bush (1989–1993), William J. Clinton (1993–2001), and George W. Bush (2001–2009). The body of reporting since 2009 has not been included in the second wave, as the presidency of Barack H. Obama is currently ongoing. A total of 608 items of reporting are distributed by presidency as follows: 22 reports under the Nixon presidency, 18 under Ford, 157 under Carter, 160 under Reagan, 79 under the first Bush, 100 under Clinton, 72 under the second Bush. The core paradigm identified as characterizing this wave is actually focused on nuclear waste: here nuclear waste appears to be a topic on its own right. This paradigm characterizes nuclear waste primarily in terms of the harm it causes, dissociated from the benefits of nuclear exploitation. The unspoken understanding appears to be that nuclear waste carries risks that cannot be eliminated, and that cleaning it up will involve costs that cannot be avoided. So instead of optimistic hope for a final and safe solution for the disposal of radioactive waste, this paradigm is preoccupied with assigning responsibility for radioactive waste.

In contrast to the wide range of disposal sites and methods contemplated during 1945–1969, land burial is the only way of disposal explored in the public discourse between 1969 and 2009. Two distinct types of radioactive waste appear to require two distinct burial methods: low-level waste, such as that coming from hospitals and research laboratories, was to be buried in several regional compacts of states; high-level waste, the byproduct of weapons and energy production, would be buried in one single national deep-geological repository. Despite significant differences between the two, in public discourse these burial methods are rather connected through concerns about justice. Public discourse focused on the equitable distribution of costs and risks associated with nuclear waste disposal as such: who should pay and who would end up bearing the risks. The heightened awareness to harm complicates the portrayal of the actors in the nuclear waste field. It turns out now that high-level nuclear waste comes not just from the national defense sources but also from the “industry” producers of energy. The federal government is charged with clearing the waste and eventually it starts charging industry for nuclear waste disposal. The plans and actions of the federal government, however, encounter increasing opposition from new actors who enter the field: state and local and tribal governments, as well as environmental organizations. These actors help shift the scientific and technological focus of public discourse away from the beneficial exploitation of nuclear reactions towards the hazards of the waste, discrediting and effectively thwarting the disposal plans of the federal government. In the following paragraphs the researcher thick-describes in more recognizably ethnographic style the public discourse on nuclear waste as represented in these second wave reports, outlining the key features of the core paradigm that dominated public discourse between 1969 and 2009 and illustrating the analysis with a number of quotations from the data.

### The Second Paradigm in Thick Description

Dissociated from benefits of nuclear exploitation, nuclear waste is now defined as pollution and primarily in terms of its harm. A January 5, 1975 report, titled “Atomic Reactors May Have to Shut,” reports that “Some nuclear reactors producing electricity in the United States may have to close down this year because of a shortage of space to store their radioactive waste, an Atomic Energy Commission Report has concluded.” A June 30, 1970 report, titled “Control of Radioactive Wastes in Sea urged at Expert’s Parley,” employs scientific terms and the notion of “poison:” “The question of pollution of ocean waters by nuclear power plants and nuclear vessels is considered particularly pressing by many of the 260 scientists and other specialists from 6 countries who are meeting here on this Mediterranean island. Their six-day conference is sponsored by an American research organization, the Center for the Study of Democratic Institutions, at Santa Barbara, Calif. ‘Every living thing on and under the sea is being poisoned with radioactive wastes,’ Dr. Jerold M. Lowenstein of the University of California Medical Center at San Francisco warned in an interview here.”

Nuclear waste carries risks that cannot be eliminated. A May 11, 1975 report, titled “One Danger of Nuclear Progress: Nuclear Waste,” makes explicit the extent of those risks: “New technologies have a way of generating new kinds of garbage which, in hindsight, often turn out to be harder to dispose of than most people dared imagine.” The same report characterizes such risks in a timeline, as having a past as well as a future. The past is introduced first: “The problem of what to do with nuclear waste–especially the most lethally radioactive ‘high level’ waste that comes from spent reactor fuel–has troubled the Federal Government for 30 years. Nuclear weapons programs, for example, have left a legacy of 79 million gallons of radioactive liquid and solid waste. More than 400 thousand gallons has leaked from storage tanks at the Government’s Hanford Reservation in Washington State since 1958.” The future of risk is characterized as lasting “millennia:” “the leakage has raised questions about the ability of human institutions to guard such dangerous materials literally for millennia.”

Nuclear waste also involves costs that give rise to disputes. These can be irregular costs, such as for cleaning up leaks. A March 25, 1979 report, titled “Paying the Price for Nuclear Waste,” tells with a bit of irony given the common knowledge of the timespan of nuclear waste decay: “Among those who live near the nuclear waste storage facility at West Valley, N.Y., there could very well be a growing feeling that by the time Federal and state officials are through haggling over who will pay for loading up the waste and disposing of it, the hazardous material stored there will no longer be hazard.” In the same spirit, the report cites the costs: “Further complicating the dispute is the fact that authorities aren’t sure precisely how they would dispose of the material or at what cost; estimates range between $200 million and $1.1 billion.” The costs involved can also be regular ones, such as for disposal. An August 30, 1992 report, titled “Wasting Away: A $1.3 Billion Tomb for Nuclear Waste in New Mexico,” names not only the costs of building the Waste Isolation Pilot Plant, or WIPP, as being $1.3 billion, but also “the high cost of maintaining the repository–nearly $500,000 a day.” Putting these costs into perspective, the report links them to the time dimension of the risk: “over the full length of its 240,000-year life, or the time that plutonium remained radioactive.”

Public discourse in this period is also very concerned with the question of responsibility: it seeks to identify the party responsible for the disposal of nuclear waste. In an October 4, 1971 report, titled: “City in Colorado Awakens to Scope of Radioactive Waste,” one Dale Hollingsworth of the local Chamber of Commerce “said the matter was clearly the Federal Government’s responsibility. ‘If I had a beach home and a sunken submarine washes up on the shore, the Government can’t say simply, ‘You get rid of that thing.’” At one point, such assignment of responsibility to the federal government, was agreeable to the federal government itself. In a October 19, 1977 report, titled “Carter Aides Offer Nuclear Waste Plan,” we learn that “[t]he Carter Administration unveiled a plan today for the Government to take over responsibility for the storage and disposal of spent fuel from atomic reactors, both in the United States and in foreign countries.” However, the federal government did not necessarily act on that responsibly. A March 5, 1980 report, titled “Rivalry in Congress Delays Up-State Nuclear Waste Cleanup,” makes this explicit: “The company that owns the tank would like to get rid of it. The State of New York wants to be relieved of its responsibility in the situation. Nearly a year ago the Carter Administration announced that it, too, agreed that the Government ought to take responsibility for what one Federal official has called a billion-dollar cleanup problem. There has been no action yet.”

Land burial is now the only way envisioned for the disposal of radioactive waste; in fact that is the case not only in the US but internationally. A May 27, 1977 report, titled: “Bonn Plans Disposal of Nuclear Wastes,” reports on the results of “two decades of experiment and debate on both sides of the Atlantic over the safest mode of nuclear waste disposal. (…) While a variety of disposal methods for high-level waste were discussed, such as launching it into space to fall into the sun, or burial beneath the sea floor, the predominant emphasis was on deposition in deep continental formations. The search for a solution is global in scope.”

Readers learn of land burial for low-level radioactive waste in the context of disputes over the burial sites. A November 11, 1984 report, titled “Northeast Faces Crisis on Radioactive Waste,” states the states’ responsibilities: “Under the Low Level Radioactive Waste Policy Act of 1980, each state is to assume responsibility as of Jan 1, 1986, for the civilian radioactive wastes produced within its borders.” The report makes clear that failing to meet such responsibilities would bear consequences: “New York, New Jersey and Connecticut, which are to be barred in 13 months from the three repositories that accept low-level radioactive wastes, have made little progress in finding alternative sites.” In the following year, a December 20, 1985 report, titled “Radioactive Waste Bill Passes,” goes into the details of the arrangement for disposal of low-level radioactive waste: “Under the compacts, several states join together and agree to share a regional disposal facility. (…) ‘This is a very firm, but fair approach to ending the intolerable burden on my state and two others have had to shoulder in being the only waste facilities in the country,’ said Senator Strom Thurmond, Republican of South Carolina, a chief architect of the compromise measure.” However, contestation over selection of the burial sites leads to a situation of little concrete action. A December 28, 1992 report, titled: “States, Failing to Cooperate, Face a Nuclear-Waste Crisis,” continues to refer to a crisis: “The problems grow from a 12-year-old Federal law that was intended to make the disposal of such [low-level radioactive] waste more equitable to the states with dump sites. But political conflict and public opposition have frustrated attempts to carry out the law.”

Burial plans for nuclear waste of high-level radioactivity also involved a search for sites. A July 19, 1976 report, titled “Nations Tackle Nuclear-Waste Disposal,” explains that “[t]he wastes, which start off as liquids so radioactive that they boil by themselves, would be concentrated into solid form–probably glass to start with–and put inside steel cannisters. The cannisters would be shipped to national repositories, expected to be deep-lying salt beds in such nations as the United States and West Germany and some form of crystalline rock in Canada.” In addition, “[t]he United States has begun exploratory drilling at a site near Carlsbad, N.M. with a view to depositing wastes there.” The actual sites would be selected from among several contestants, as explained in an April 16, 1978 report titled “Government Problems in Finding Disposal Sites for Nuclear Wastes May Delay Building of Power Plants:” “The Government is focusing on burying nuclear waste in deep geological formations of salt, granite or shale, as it looks for permanent disposal sites. (…) Although the Government initially announced that it was looking at 36 states for sites, the search has apparently been narrowed to nine.” Some years later, from an August 16, 1985 report titled “New Rules on Radioactive Waste,” we learn that “waste materials from nuclear reactors and atomic energy defense facilities” are in effect lumped together and that the search had been narrowed to just three: “The Energy Department has identified three potential sites for the first disposal facility: Hanford, Wash., Deaf Smith County, Tex., and Yucca Mountain, Nev.” Eventually, from a February 16, 2002 report titled “Bury the Nation’s Nuclear Waste in Nevada, Bush Says,” we learn that now only one site is being considered: “President Bush said today that a 57-year accumulation of nuclear waste from power plants and weapons should be buried in the Nevada desert at Yucca Mountain, declaring that an end to the 40-year search for a place to isolate radioactive waste was necessary to ‘protect public safety, health and the nation’s security.’” However, a report from February 17, 2008, titled “As Nuclear Waste Languishes, Expense to U.S. Rises,” indicates that actual burial is still to happen: “Forgotten but not gone, the waste from more than 100 nuclear reactors that the federal government was supposed to start accepting for burial 10 years ago is still at the reactor sites, at least 20 years behind schedule. (…) Yucca has been beset with legal and managerial problems, and it is not clear whether the geology is suitable for the goal, storing the waste for a million years with only very small radiation doses for people beyond the site boundary.”

Discourse on waste of both high-level and low-level radioactivity is permeated by the concern for justice: the question really is who would carry the costs and who would bear the risks. In the context of storage risks, a February 24, 1985 report, titled: “A Time of Decision Nears on Nuclear Waste,” quotes Representative Harriet Keyserling of South Carolina: “Enough is enough and fair is fair. (…) We’ve done our fair share; it’s time for others. (…) If there are risks, they ought to be shared.” A September 16, 1991 report, titled “Connecticut Confronts Being Its Own Nuclear Waste Site,” brings up the same theme of equity in the context of storage costs: “Environmentalists say that keeping nuclear waste in the state where it was produced is probably more fair, but they agree that it is a harsh sort of justice. ‘It is a very healthy principle that those who benefit from a technology should pay the full costs,’ said Fred Millar, director of the toxics project at Friends of the Earth, a Washington-based environmental group.”

It turns out that high-level waste comes not just from national defense, but also from the industry producers of energy. Several reports indicate that, consequently, in accordance with the “polluter pays” principle, industry should be made to pay for the disposal. In a September 12, 1982 letter to the editor, titled “Very Real Progress on Nuclear Waste,” Secretary of Energy James B. Edwards explains: “Nuclear waste is a bipartisan matter, the settlement of which is in the interest of all Americans. Between now and the year 2000, under the pending legislation, utilities will make advance payments totaling up to $25 billion to fund the entire cost of our national program well into the next century. There will be no Federal expenditures.”

The actors in the nuclear waste field, and especially their mutual interactions, receive much attention in the second wave of public discourse. In contrast to the first paradigm, the second paradigm conceptualizes the US federal government as but one of the actors in the field, albeit a key actor. The federal government is viewed primarily in its governing function, specifically in managing radioactive waste and risks. For example, a January 30, 1971 report, titled “Radioactive-Waste Repository Planned,” names the Atomic Energy Commission and tells of federal “plans to develop in Kansas a storage facility for radioactive wastes big enough to meet the nation’s needs for the rest of the century.” The actions of the federal government include studying the problem of nuclear waste as well as, increasingly, articulating the federal position vis-a-vis nuclear waste. A May 16, 1971 report, titled “Radioactive Ore Waste Stirs Fears,” informs of EPA studies: “Paul Smith of the Denver radiological office of the United States Environmental Protection Agency said this week that he was drawing up a proposal for studies in all affected states. (…) He said the EPA was already studying the problem in Riverton, Wyo.” In an October 4, 1971 report, titled “City in Colorado Awakens to Scope of Radioactive Waste,” the Atomic Energy Commission articulates its position on “tailings,” a kind of radioactive waste, namely radioactive sand, used as building material: “At the present time, we find it difficult to conceive of any mechanism whereby the radioactive material which is now so widely dispersed could become so concentrated as to exceed current applicable standards for protection against radiation.”

However, and increasingly, the federal government’s actions are portrayed as failed actions or as wrong actions. A December 10, 1988 report, titled “Blowing, Flowing and Crawling, Nature is Spreading Nuclear Waste,” illustrates this: “The Energy Department’s reports, covering 12 of the 17 principal weapons factories and laboratories, show that radioactive waste was handled in careless and archaic ways that would have been illegal in private industry.” In fact, the federal government now faces fierce opposition. Only two days after the report on the federal plans for the Kansas facility quoted above, on February 17, 1971, a report titled “Kansas Geologists Oppose A Nuclear Waste Dump” informs readers of opposition from Kansas: “‘The fact is,’ Representative Skublitz wrote the Governor in a letter that arrived in Topeka this afternoon ‘that however the Atomic Energy Commission may phrase it semantically, a part of Kansas is proposed as a dump for the most dangerous garbage in the knowledgement of mankind. A dump is a dump not matter how the garbage is packed.”

Other actors appearing in their interaction with, and namely opposition to, the federal government include the states. A December 20, 1984 report, titled “U.S Names 3 Sites for Atomic Study,” informs readers of government plans and state opposition in the same report: “The Government chose three areas in Texas, Nevada and Washington today as the leading candidates to become a permanent site for disposing highly radioactive nuclear wastes. (…) The Governors of Texas and Nevada promised to oppose dumping the waste in their states.” States can oppose both the federal government and the nuclear industry. A January 28, 1970 report, titled “Congressmen Clash with Minnesota Governor,” illustrates the former: “Gov. Harold LeVander of Minnesota and two members of the Congressional Joint Committee on Atomic Energy Clashed today over Minnesota’s asserted, but not established rights to set more rigid limits on radioactive discharges from nuclear power plants than the Atomic Energy Commission requires.” A February 1, 1978 report, titled “California Board Won’t Give Permit for New Nuclear Energy Project,” illustrates the latter: “Atomic power is encountering an increasingly rocky road in California. The State Energy Commission recommended to the Legislature last week that it not grant a permit to the San Diego Gas and Electric Company to process with its $3 billion Sundesert nuclear-generating project, near Blythe on the Colorado River.” A July 28, 1978 report, titled “Carey Seeks to Bar New Power Plants Using Nuclear Fuel,” shows that similar action is taken by other states: “Governor Carey’s administration will seek to bar construction of any future nuclear power plants in New York State because of a lack of capacity to handle radioactive waste, the State Energy Commissioner said today.”

A significant front of such opposition is the transportation of nuclear waste. An August 25, 1979 report, titled “Two North Carolina Ports Closed to Nuclear Wastes,” shows a state’s power to stall action at the international level: “State officials have closed the ports at Wilmington and Morehead City to shipments of nuclear waste from overseas.” Clearly, such opposition is political in nature. An August 3, 1980 report, titled “Report Urges Regional Plan on Radioactive Waste Sites,” quotes Governor Bruce Babbitt of Arizona: “[T]he problem is political and involves all the problems of federalism, with none of the 50 states likely to want a dump within their borders.”

Local governments are actors in this field too; they can oppose plans from the federal level. A March 7, 1976 report, titled “Moving Nuclear Waste,” informs of such action by New York City: “Until several months ago, Brookhaven [National Laboratory] was able to truck the nuclear waste, or ‘soup,’ west through New York City then south to a Federal processing plant on the Savannah River, near Aiken, S.C. But the city’s Health Commissioner, Dr. Lowell E. Bellin, fearful of any mishaps, ordered a ban on such shipments through city streets.” A March 17, 1978 report, titled “South Jerseryans Win U.S. Hearing on Banning Atomic Waste Storage,” tells of another instance: “South Jersey residents who fear that Salem County will become a major storage center for highly radioactive nuclear fuel wastes expressed gratification today that they had won their battle for a public hearing on the issue.” The town’s reported concern was articulated in these terms: “The spent fuel, stored in water inside concrete, will be radioactive for up to 500,000 years. Contact with it could cause cancer, genetic disorders and other illnesses.” A January 1, 1979 report, titled “Rockland Seeks Way to Restrict Nuclear Wastes,” indicates that similar banning was pursued more widely: “Following reports that radioactive wastes and materials from atomic reactor plants in the Northeast were being shipped regularly through Rockland County to processing areas in the South and West, the county’s officials are considering following the lead of New York City in severely limiting such shipments.”

At times, a mayor might express ambivalence, as illustrated by a March 9, 1981 report titled “Jersey Reactor Creates Tax Gains And Radioactive Waste Problem:” “Officials speak highly, on the one hand, of their giant nuclear neighbor and the money and jobs it has brought in; on the other, they regard the accumulating wastes with some uneasiness. ‘I think you could say there is a sort of conflict there, yeah,’ said the town’s part-time Mayor, Samuel E. Donelson.” Some municipalities might even pursue the federal benefits associated with making available land for disposal. A March 8, 1970 report, titled “Nerve Gas Offer Leading to Atomic Waste Deal,” tells of “Mayor LaRue [who] promised a warm welcome in his constituency (…) Mayor LaRue and county spokesmen affirmed their willingness to accommodate ‘pariah’ industry in their high plateau’s wide open wheat and cattle country. The county has fewer than 4,000 residents.” A May 16, 2004 report, titled “Proposal for Nuclear Waste Train Splits a Nevada Town,” features “Mayor Kevin Philips [who] has figured out a way to lift the fortunes of his struggling hamlet tucked in the mountains about 130 miles north of Las Vegas” by offering his town “to be the transfer station for the nation’s spent nuclear fuel that is supposed to be stored in the Yucca Mountain repository beginning in 2010.”

In the 1990s, a number of reports explore the role of tribal governments in the action involving nuclear waste disposal. In an October 17, 1991 report, titled “Idaho Tribe Stops Nuclear Waste Truck,” informs of one tribe in conflict over the transportation and storage of radioactive waste: “The spent fuel from a plant in Colorado was being taken to an Energy Department weapons plant near Idaho Falls. The 12-hour confrontation between the tribal police, the truck driver, a Federal inspector, the state police, and representatives of the utility occurred along Interstate 15 in southeastern Idaho.” The reason for the incident was that the tribe sought to prevent the truck from passing through the reservation, echoing the efforts of cities and states to ban the transportation of nuclear waste through their territories. Tribal governments could also appear as willing to store nuclear waste on tribal territory, echoing once more the actions of certain municipalities. An August 6, 1993 report, titled “Tribe on Path to Nuclear Waste Site,” informed that “[a]n Indian tribe said yesterday that it wanted to begin ‘credible, formal discussions’ with the Federal Government about storing high-level nuclear waste for at least a few years on its reservation in New Mexico. (…) In exchange for accepting the waste, the tribe wants tens of millions of dollars a year and might also ask for things like roads or water and sewage plants.”

Environmental organizations appear as another important player in the nuclear waste field. In a January 12, 1979 report, titled “How the Critics Responded to the Nuclear Waste Report,” the Sierra Club is introduced as the agent that articulates the concerns in the conflict over West Valley, New York: “The Sierra Club, a national organization in the environmental field, contends that New York State, which owns the grounds around the plant, has at most collected 8 cents a cubic foot, or $192,000, from the users of the burial grounds. The club says that if it the state pays out $110,000 in capital costs and $40,000 a year for surveillance for the estimated 1,000 years of toxicity, total costs to state taxpayers will be more than $1 million, or five times the amount collected.” Environmental organizations often also take legal action. For example, following up on this same West Valley, New York case, a July 11, 1982 report titled “U.S. and State Nearing Solution on Nuclear Waste Pile” shows the Sierra Club in such action: “The Sierra Club has brought legal action to try to prevent the company from leaving so much of the cost to the state and Federal governments.” Environmental organizations also employ scientific investigation to highlight the long-term hazards of radioactive waste. A January 15, 1982 report, titled “Agency may alter Atom Waste Policy,” quotes the representatives of two such environmental organizations: “John Hinck, a spokesman for Greenpeace, an environmental group, said that the scuttled vessels would still emit high radioactivity (…) Thomas Cochrane of the Natural Resources Defense Council, an environmental group, said there were few data on long-range effects of nuclear waste dumping.”

The cumulative effect of such action was that disposal plans of the federal government were often discredited and effectively thwarted. A November 29, 1989 report, titled “U.S. will start over again on planning for Nevada Nuclear Waste Dump,” exposes such failure: “The Government has abandoned a twenty-year effort costing $500 million to plan the nation’s only dump for highly radioactive nuclear waste because it lacks confidence in its work thus far, officials said last night. It has decided to start over. (…) Three decades after the beginning of the use of nuclear power for electricity, the Government still has no repository for the spent fuel.”

### Why are there these Two Paradigms?

Why are there these two paradigms? Why is it that between 1945 and 1969 nuclear waste in the United States was conceptualized in the ways and words illuminated through this research? And why is it that between the years 1969 and 2009 that very same natural and physical phenomenon was conceptualized in the strikingly different ways also outlined in this paper? Why this particular morphing or paradigm shift? Did it happen because of key historical events such as the accidents at Three Mile Island or Chernobyl? Was it because of the Vietnam War or the Cold War or the ending of the Cold War? Was it because of the presidents and their policies? Did Eisenhower have more of an impact than Truman? Did Regan have more of an impact than Carter? Or, instead, were the policies of these presidents rather impacted by the public discourse? Or were the dynamics of public discourse due to other technological or political or societal shifts of which the six or seven decades at the focus of this study saw many: the civil rights movement, the peace movement, the women’s movement, the broader environmental movement, and more? Or, indeed, did these paradigms help bring such shifts to their particular empirical shapes?

Such important questions of causality are often addressed by the nuclear waste scholarship, including the literature discussed earlier in this paper. Certain correlations are not too difficult to establish, and proposing certain causalities can be tempting, including to this author. It can be just as tempting to argue against commonly proposed causalities. For example, whereas the 1979 accident at Three Mile Island is commonly assumed as causal to the understanding of nuclear waste primarily in terms of its hazards, this project empirically places the emergence of the “political economy” discursive wave in 1969: we thus learn that public discourse had been conceptualizing nuclear waste in terms of risks and harms for a decade before the Three Mile Island accident happened.

In any event, in delineating the discourse on nuclear waste as the object of an empirical though qualitative study, and in separating nuclear waste the sociocultural phenomenon from nuclear waste the material phenomenon as well as from the common or contemporary cultural understandings thereof, the author recognizes that the empirical data of the present research simply do not provide grounds for addressing with empirical certainty the question of why are there these two paradigms. Keeping with the project’s empirical ethos, the author reckons that establishing any of the candidate lines of causality enumerated above, or indeed any others, calls for data that are beyond the scope of what was investigated here.

While readers will decide for themselves whether to count this empirical ethos as a shortcoming or as a strength, the author believes that one of the present study’s key values, besides the articulation of the finding of the two paradigms, is the rigorous documentation of nuclear waste in its sociocultural existence. Identifying and establishing causalities will hopefully become the focus of future research and interpretations as well as attract the efforts of other scholars to build on the empirical findings outlined in this paper.

## Conclusions

This project set out to illuminate nuclear waste as a sociocultural phenomenon in the United States through an anthropological approach. The key finding is that, between 1945 and 2009, American public discourse captured and framed nuclear waste from the angles of two paradigms that conceptualized nuclear waste in strikingly distinct ways and cultural terms.

The first paradigm, corresponding to the 1945–1969 wave of American public discourse on nuclear waste that peaked under President Eisenhower, was optimistic and future-oriented; it was absorbed in the beneficial utilization of nuclear power for peace, energy production, and prosperity. Nuclear waste was recognized as a peace-time problem that would meet a solution in the near future. That solution would come from scientific discovery and technological progress. In the second paradigm, corresponding to the 1969–2009 wave of American public discourse on nuclear waste that peaked under President Carter, nuclear waste appeared to be more of an issue in its own right. As such, nuclear waste was conceptualized as harm inherited from the past that needed to be cleaned up. Public discourse sought to identify the party responsible for such decontamination in terms both economic and of risk.

Another important difference between the two paradigms concerns the manner of actual disposal of material waste. The first paradigm looked at a variety of ways of disposal: burning, burial, flushing into sewage, dumping into the ocean. Even ideas such as blasting nuclear waste into outer space were entertained. Each such solution to the nuclear waste problem was conceptualized as final; however, eventually awareness grew that none of these methods would be sufficient for preventing the harm caused by radioactivity. By contrast, the second paradigm focused solely on containment, even temporary containment, and it distinguished two categories of nuclear waste: waste of low-level radioactivity, for which burial was conceptualized as acceptable if not desirable; and high-level radioactive waste for which deep-burial was sought and contested.

An additional difference concerns the actors involved in conceptualizing nuclear waste. Action in the first paradigm was carried out by the US government, which, with its scientists, researchers, and inventors, was envisioned in competition with foreign nations. The second paradigm identified different governing bodies within the United States: the federal government and its agencies, various state governments, local governments, and tribal governments, often in conflict with each other regarding the topic of where to dispose of nuclear waste. Eventually, public utilities, private industry, citizen action groups, and environmental organizations also appeared as parties in legal conflicts.

Finally, besides these articulations and their chronological timing, the study’s value to future scholarship lies with the empirical documentation of nuclear waste in its sociocultural existence in the paper’s “thick description” sections.
